# Meta-heterogeneity: Evaluating and Describing the Diversity in Glycosylation Between Sites on the Same Glycoprotein

**DOI:** 10.1074/mcp.R120.002093

**Published:** 2020-12-08

**Authors:** Tomislav Čaval, Albert J.R. Heck, Karli R. Reiding

**Affiliations:** 1 Biomolecular Mass Spectrometry and Proteomics, Bijvoet Center for Biomolecular Research and Utrecht Institute for Pharmaceutical Sciences, University of Utrecht, Utrecht, the Netherlands; 2 Netherlands Proteomics Center, Utrecht, the Netherlands

**Keywords:** Meta-heterogeneity, Micro-heterogeneity, Macro-heterogeneity, Glycan, Glycosylation, Proteoforms, Glycoproteoforms, Plasma/serum glycoproteins, Immunoglobulins, Acute phase proteins, ApoB-100, apolipoprotein B-100, CID, collision-induced dissociation, ECD, electron-capture dissociation, EPO, erythropoietin, ETD, electron-transfer dissociation, Fab, fragment antigen-binding, HCD, higher-energy collision dissociation, MPO, myeloperoxidase, *O*-Man, *O*-mannosylation

## Abstract

Mass spectrometry–based glycoproteomics has gone through some incredible developments over the last few years. Technological advances in glycopeptide enrichment, fragmentation methods, and data analysis workflows have enabled the transition of glycoproteomics from a niche application, mainly focused on the characterization of isolated glycoproteins, to a mature technology capable of profiling thousands of intact glycopeptides at once. In addition to numerous biological discoveries catalyzed by the technology, we are also observing an increase in studies focusing on global protein glycosylation and the relationship between multiple glycosylation sites on the same protein. It has become apparent that just describing protein glycosylation in terms of micro- and macro-heterogeneity, respectively, the variation and occupancy of glycans at a given site, is not sufficient to describe the observed interactions between sites. In this perspective we propose a new term, meta-heterogeneity, to describe a higher level of glycan regulation: the variation in glycosylation across multiple sites of a given protein. We provide literature examples of extensive meta-heterogeneity on relevant proteins such as antibodies, erythropoietin, myeloperoxidase, and a number of serum and plasma proteins. Furthermore, we postulate on the possible biological reasons and causes behind the intriguing meta-heterogeneity observed in glycoproteins.

## Highlights

• Micro- and macro-heterogeneity describe variation and occupancy of glycan sites.
• We propose meta-heterogeneity: glycan variation across multiple sites of a protein.
• Many glycoproteins exhibit meta-heterogeneity, including immunoglobulin G.
• Study of meta-heterogeneity will be critical to understand glycoprotein function.

## Graphical Abstract

**Figure:**
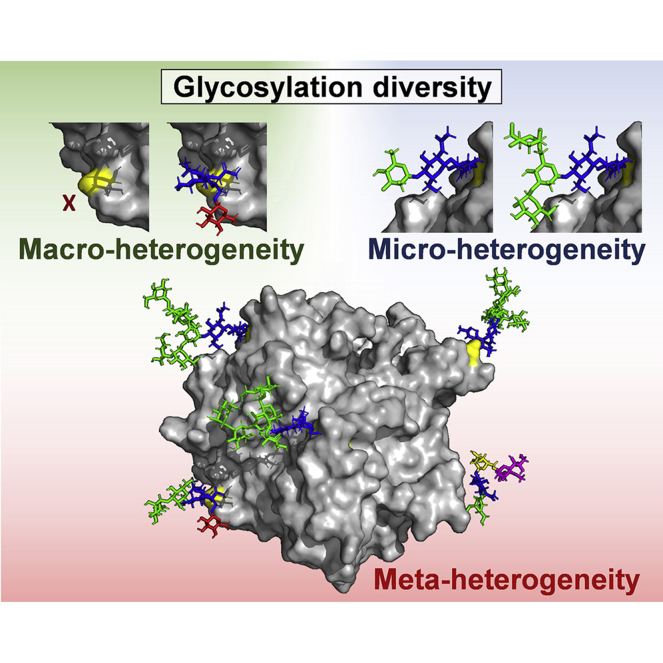

## In Brief

Diversity in protein glycosylation can be described in terms of micro-heterogeneity and macro-heterogeneity, respectively, referring to the variation and occupancy of glycans at a given glycosylation site. However, these terms are not sufficient to describe a higher level of regulation when proteins are multiply glycosylated. For this, we propose the term meta-heterogeneity: variation in glycosylation across multiple sites of a given glycoprotein. In this review, we describe several remarkable examples of glycoprotein meta-heterogeneity and underline the need for its investigation.

**Fig. 1:**
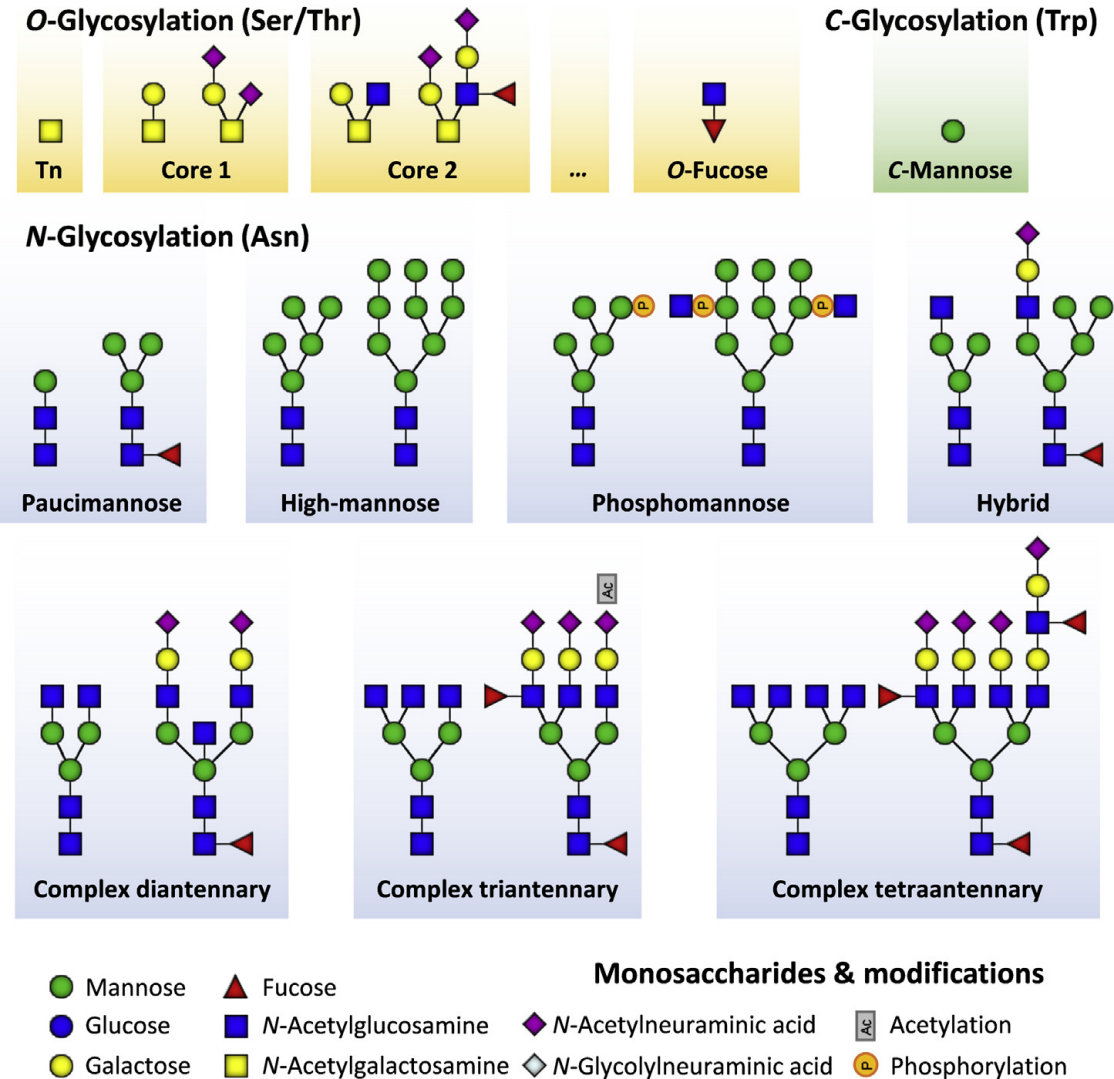
**Overview of the glycosylation features discussed in this review that contribute to meta-heterogeneity**. Glycan compositions were chosen to represent a small and a large variant within the given group (*e.g.*, high-mannose). Tn, Core 1, and Core 2 *O*-glycosylation represent only a selection of the large group of possible *O*-glycan core structures, as the compositions themselves can be much larger. *O*-Acetylation has been positioned at a triantennary glycan but may in principle occur at any sialic acid (*N*-acetylneuraminic acid or *N*-glycolylneuraminic acid).

Glycosylation is by far the most abundant posttranslational protein modification encountered in nature. Glycosylation plays critical roles in all facets of health and disease, and slight changes in the structure of the glycan attached to the polypeptide backbone can have a dramatic biological effect (1, 2, 3). Although often simply regarded as a uniform modification, it actually encompasses dozens of different types of glycosylation with new ones discovered regularly (Fig. 1) (4, 5, 6, 7).

Perhaps the most well-known and certainly the most studied type of glycosylation refers to *N*-linked protein glycosylation, or *N*-glycosylation. Here, a single uniform precursor consisting of two *N*-acetylglucosamines (GlcNAc), nine mannoses (Man) and three glucoses (Glc), in short Glc_3_Man_9_GlcNAc_2_, is transferred cotranslationally to a side chain of asparagine in an Asn-Xxx-Ser/Thr sequon (where Xxx can be any amino acid besides proline). In addition, *N*-glycans may in some cases be transferred to an Asn-Xxx-Cys/Val sequon, but this appears to result in relatively low degrees of occupancy.

A second widely encountered type of glycosylation is the so-called mucin-type *O*-glycosylation, in which a single *O*-GalNAc residue is transferred to a hydroxyl group of threonine, serine, or tyrosine (8, 9, 10, 11). Unlike *N*-glycosylation, in which a single precursor is transferred to the polypeptide backbone, initiation of *O*-glycosylation can be catalyzed by at least 20 distinct *O*-GalNAc transferases, which have both overlapping and differential substrate preferences (12, 13, 14). Initiating *O*-GalNAcs can also be further extended to create more elaborated *O*-glycan structures, common examples including Core 1 (Galβ1,3-GalNAc) and Core 2 (Galβ1,3-[GlcNAcβ1,6-]GalNAc) type *O*-glycans. *O*-Glycans mainly serve to stabilize the protein structure and protect against proteolytic cleavage (15), but they are also implicated in various pathophysiological states such as IgA nephropathy (16), Tn-syndrome (17), impaired leukocyte recruitment (18), and tumorigenesis (19).

In addition to the mucin type *O*-glycosylation, there are also numerous other classes of *O*-glycosylation that have received so far less attention (9, 20). Examples include *O*-fucosylation (21, 22, 23) and *O*-glucosylation (24, 25, 26) found on epidermal growth factor-like and thrombospondin repeat domains, *O*-galactosylation (27, 28) on collagen domain hydroxylysines, and *O*-mannosylation (*O*-Man) mainly associated with the alpha-dystroglycan and cadherins (29). Although the aforementioned protein modifications are never found in the nucleocytoplasmic space, *O*-GlcNAcylation, catalyzed by the *O*-GlcNAc transferase, can be found in nuclear, cytoplasmic, and mitochondrial compartments (30). In contrast to the other types of modifications, *O*-GlcNAcylation is a dynamic modification that is thought to serve, *e.g.*, as a nutrient sensor regulating transcription, signaling, mitochondrial activity, and cytoskeletal functions (31). Similar to the nucleocytoplasmic *O*-GlcNAcylation that is widespread in eukaryotes and bacteria, yeast appear to instead direct that role to nucleocytoplasmic *O*-mannosylation (32).

Finally, besides the above-mentioned *N*- and *O*-linked glycosylation, there also exists *C*-mannosylation occurring on tryptophan residues within the Trp-Xxx-Xxx-Trp motif. *C*-Mannosylation is a rare and relatively unstudied modification involved in protein folding and the stabilization of thrombospondin repeats (33). In all, these examples underline the extreme diversity of modifications that are encompassed under the singular term glycosylation.

## Contemporary methods for glycosylation analysis

Over the last decade, there has been a surge of technological developments in the field of protein glycosylation analysis. We witnessed developments of methods for large-scale glycosylation analysis such as lectin microarrays, in which lectins that recognize specific glycan epitopes are printed on a glass slide and interrogated with fluorescently labeled analytes (34, 35). The resulting patterns give insight into the glycosylation profiles of a tested biological system and have enabled the discovery of glycan biomarkers for inflammatory bowel disease (36), esophageal cancer (37), liver fibrosis (38), and melanoma (39). Another elegant approach uses the chemical release of all glycans from a sample, *e.g.*, lung tissue, and immobilizes these released glycans on a glass slide (40). These so-called shotgun glycan arrays have, for instance, enabled a good overview of the influenza A virus receptors (41), discovering that, aside the well-known α2,6-linked sialylated glycans, the viruses also strongly recognize phosphorylated, nonsialylated *N*-glycans (41).

In addition to these array-based methods for glycan characterization, there are also numerous others based on liquid chromatography and capillary electrophoresis (42, 43, 44, 45, 46, 47). Currently by far the most popular approach, also used in the most of studies mentioned earlier, are mass spectrometry (MS) based. MS enables a fast and sensitive readout of the glycosylation status with minimal sample requirements. It can be performed as a stand-alone technique or in combination with liquid chromatography and capillary electrophoresis to gain an even deeper understanding of the glycan structure (48, 49, 50, 51). In terms of approaches for glycosylation analysis, MS can be divided in three distinct levels: (1) determination of released glycan profiles, (2) determination of modified sites and their accompanying structures through the analysis of glycopeptides, and (3) direct analysis of intact glycoproteins that enables a global view of the protein glycosylation status (52). Over the last few years, we have witnessed a storm of events that have made MS-based glycoproteomics amenable to high throughput and accessible to the wider scientific community. This can be broadly ascribed to developments in three different areas: the improvement of glycopeptide enrichment methods, the improvement in glycopeptide fragmentation methods and data acquisition strategies, and, finally, the improvement in software solutions for automated glycopeptide identification.

## Glycopeptide enrichment methods

To analyze glycopeptides, ideally one first enriches them from the vast pool of nonmodified peptides. Perhaps the most common approach for glycopeptide enrichment is the use of specific glycan-binding proteins called lectins. Currently there are hundreds of commercially available lectins with differing glycan-binding specificities. For *N*-glycans the most common ones include wheat germ agglutinin, concanavlin A, and *Aleuria Aurantia* lectin. Wheat germ agglutinin recognizes both GlcNAc and NeuAc residues, making it a popular choice for the characterization of complex *N*-glycopeptides (53). Next, concanavlin A recognizes mannose residues and is typically used for the enrichment of high-mannose, hybrid, and diantennary N-glycans (54). *Aleuria Aurantia* lectin is a lectin that is used for its strong recognition of core-fucosylated *N*-glycans, which are often increased in abundance in cancerous states (54, 55). Although lectins are very useful for specific applications, it has to be noted that their binding specificities make comprehensive enrichment of *N*-glycans a challenging affair (54). To address this, other enrichment methods based on general *N*-glycan properties have been developed. Popular examples include hydrophilic-interaction liquid chromatography, in which enrichment is based on the increased hydrophilicity of glycopeptides in comparison with nonglycosylated peptides, and strong anion exchange, in which enrichment is based on negatively charged sialic acids (48, 56, 57, 58). Finally, a third way to enrich glycopeptides is to chemically modify the glycan portion to make it more amenable for enrichment (59). One example of this approach is based on metabolic labelling of the glycoproteome by utilizing azido-labeled sugars, which are utilized by biosynthetic machinery and incorporated into various glycans (60). This approach can furthermore be combined with chemical enrichment and isotopic recoding of glycopeptides (61).

## Glycopeptide fragmentation and data-acquisition strategies

A significant challenge in the analysis of intact glycopeptides by MS is to transform the *m/z* values of each analyte into an unambiguous identification. For general proteomics on nonmodified peptides this is a well-established approach whereby each peptide ion is isolated and subjected to fragmentation by collision-induced dissociation (CID). CID preferentially breaks peptide bonds resulting in a series of so-called *b*-ions (N-terminal fragment) and *y*-ions (C-terminal fragment) from which a sequence of the fragmented peptide can be determined. In addition, fragmentation spectra can be compared with databases for automated and highly confident identification of peptides and proteins they are derived from. In contrast, CID of glycopeptides mainly produces sequential cleavages of glycan monosaccharides alongside the glycosidic bond. Although this method is useful for structural information, it is very challenging to identify the underlying peptide sequence of the fragmented glycopeptides. To address this challenge, other fragmentation methods more amenable for intact glycopeptide analysis are needed.

Electron-capture dissociation (ECD) was one of the early alternative fragmentation methods showing great promise for the characterization of intact glycopeptides (62). In this approach, low-energy electrons irradiate glycopeptide cations that upon capturing an electron will undergo fragmentation. In ECD, *c*- and *z*-ions are observed, which arise from the N-Cα bond cleavage of the peptide backbone. Glycopeptides analyzed with ECD will retain their intact glycan attached to the peptide backbone, whereas series of *c*- and *z*-ions enable the identification of the modified peptides. However, ECD is usually only available on Fourier-transform ion cyclotron resonance mass spectrometers that are not widely available owing to the extremely high costs associated with the acquisition and maintenance of such instruments. In addition, in ECD only around 20% of the analyzed glycopeptides undergoes effective fragmentation, whereas the rest is usually only charge reduced without dissociation (63). The availability of electron-based methods was substantially enhanced through the introduction of electron-transfer dissociation (ETD) fragmentation (64), which can be more readily implemented to ion trap–containing mass spectrometers. In this case, instead of a cloud of low-energy electrons, reagent anions such as fluoranthene are used to collisionally transfer an electron to the analyte.

The combination of CID/ETD fragmentation proved to be a strong approach for the characterization of single glycoproteins (63). However, owing to the somewhat slower speed of ETD, and the fact that in complex mixtures even the best enrichment approaches still result in a significant proportion of nonglycopeptides, early forays into intact glycopeptide analysis of the complex mixtures resulted in a very limited number of identifications. A solution to this issue came from the detection of highly abundant oxonium ions formed from glycopeptides during beam-type CID experiments (65). These had generally not been observed with conventional CID-based ion-trap analyses (owing to limited trapping efficiency in the low *m/z* range) but proved readily observable by triple quadrupole instruments equipped with beam-type CID (66). The most commonly formed oxonium ions are derived from HexNAc (*m/z* 204.0867 [M + H]^+^), Hex (*m/z* 163.0601), Neu5Ac (*m/z* 292.1027 and 274.0921), HexNAcHex (*m/z* 366.1395), HexNAcHexFuc (*m/z* 512.1974), and HexNAcHexNeu5Ac (*m/z* 657.2349). The detection of glycopeptide-specific oxonium ions, coupled with the high mass accuracy provided by the Orbitrap instruments, was used to create smart acquisition strategies. In these, ETD fragmentation would be triggered only upon the observation of signature ions during higher-energy collision dissociation (HCD), which, akin to beam-type CID, also resulted in the formation of abundant oxonium ions (67, 68, 69, 70). This could be considered as a second level of glycopeptide enrichment where enrichment occurs within the mass spectrometer itself.

Further improvements in the field of intact glycopeptide characterization were enabled by the so-called hybrid fragmentation methods, in which, *e.g.*, HCD and ETD are combined into a single method such as EThcD (71). In EThcD, glycopeptides undergo ETD fragmentation followed by HCD fragmentation of both the ETD precursor and product ions. In this way improved sequence coverage and site localization of glycopeptides are achieved when compared with HCD or ETD fragmentation alone (72, 73, 74, 75). Another example of a hybrid approach is stepped-HCD in which the precursor is exposed to a number of (*e.g.*, three) different HCD energies, the resulting mass spectrum being a sum of ions from the different energies (76, 77). In this way, each spectrum contains sequential sugar losses, enabling glycan annotation (low-energy HCD) and peptide backbone fragments (high-energy HCD). Finally, it is worth nothing that with these two hybrid fragmentation approaches (stepped HCD and EThcD), large-scale studies characterizing thousands of intact glycopeptides have become feasible (72, 78, 79).

## Software solutions for automated glycopeptide identification

Although we are able to measure and sequence thousands of glycopeptides, their confident identification still represents a major hurdle toward a truly systems biology approach of the glycoproteome. Although standard proteomics approaches have a wealth of software solutions for identification, quantitation, and comparison of data, glycoproteomics still lags substantially behind. The reason lies in the difficulty in controlling the false discovery rates for both the peptide and the attached glycan simultaneously (80). Over the years, dozens of software solutions have been published for intact glycopeptide analysis (81, 82). However, a large proportion of them are only useful in specific cases developed for and require bioinformatics expertise to use. Furthermore, a recent study found that around 30% of published software resources are no longer available owing to lack of maintenance following the publication, and up to 50% of software resources are deemed as “difficult to install” (83).Therefore, it may not be surprising that the vast majority of published glycopeptide studies used two of the best-maintained software solutions: pGlyco2.0 and Byonic (84). Byonic is capable of analyzing both the HCD and EThcD data and is specifically developed with intact glycopeptide analysis in mind. On the other hand, pGlyco2.0 (78) identifies intact glycopeptides based on stepped-HCD data, and although at present it does not support EThcD data, developments are underway to address this issue. A third well-supported and freely available software solution handling both HCD and EThcD glycopeptide searches is ProteinProspector (53, 85, 86), although it has so far been less often employed than the previously mentioned software. In addition, some general proteomics search engines such as MSFragger and MetaMorpheus have an ongoing development to include the support for intact glycopeptide analysis (87, 88).

## Meta-heterogeneity

**Fig. 2:**
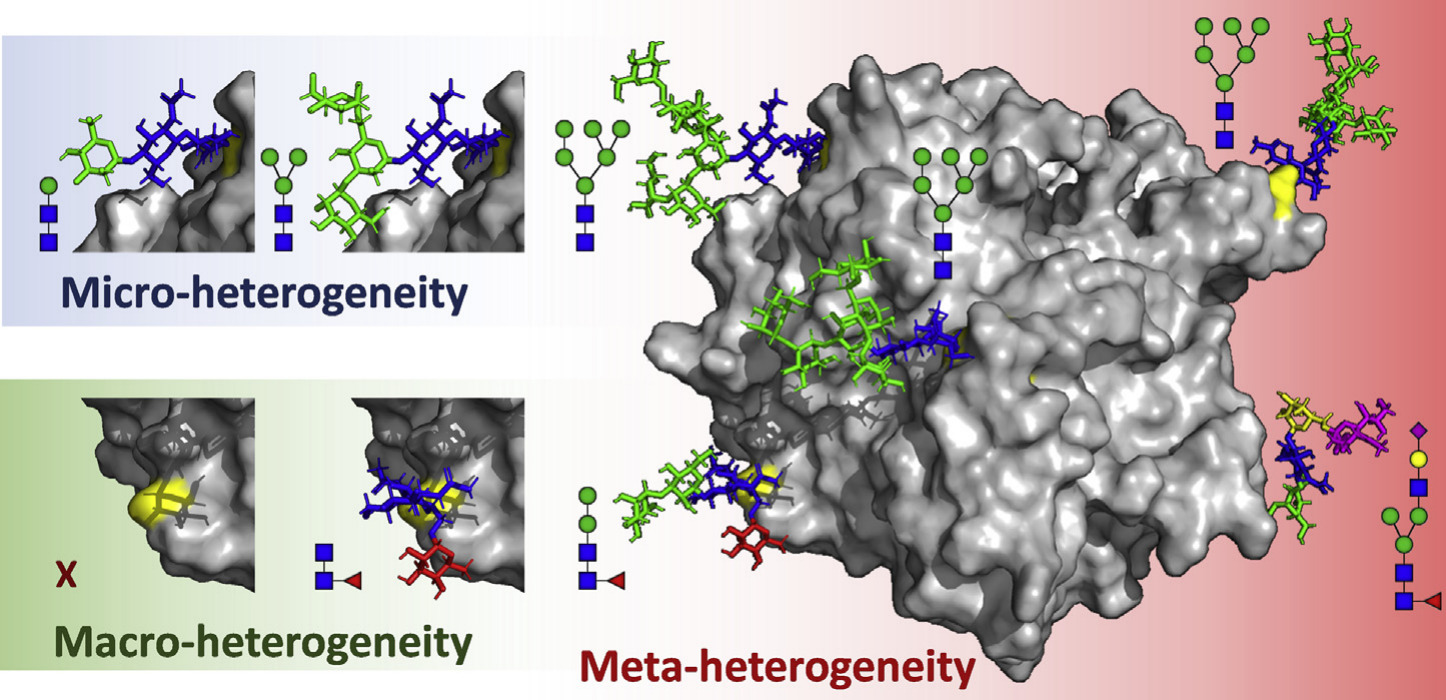
**Defining meta-heterogeneity**. Although micro-heterogeneity (*top left*) describes the variation in glycan species at a given glycosylation site and macro-heterogeneity (*bottom left*) describes to which degree a glycosylation site is occupied, these terms are not sufficient to describe a higher order of regulation across a glycoprotein with multiple glycosylation sites. As such, we propose the concept of meta-heterogeneity (*right*) to describe the variation in glycosylation across all glycosylation sites of a given protein. In the chosen example (myeloperoxidase monomer; 1CXP) (90, 91) it can be seen that the *N*-glycosylation sites (*yellow residues*) are decorated by very different glycans, ranging from paucimannose to hybrid-/complex-type. This difference makes for a protein with high glycan meta-heterogeneity, indicating selective regulation and highlighting potential differences in structure–function relationships.

As glycoanalytical methods have evolved to reveal protein glycosylation in a site-specific manner (*e.g.*, by bottom-up mass spectrometry) and how glycan species co-occur on a given protein (*e.g.*, by intact/native mass spectrometry), it has become apparent that sites can be individually regulated. Macro-heterogeneity describes the variation in glycosylation site occupancy and micro-heterogeneity the variation in glycan species, but these are not sufficient to describe what is happening on the whole protein (89). Therefore, we here propose the term meta-heterogeneity to describe a higher level of glycan regulation: the variation in glycosylation across multiple sites of a given protein (Fig. 2). The prefix meta- originates from the Greek “*μετά*-,” which can be translated as “after” or “beyond” and is generally used to make a concept more encompassing or overarching. The choice of language pays tribute to the Greek origins of micro- (*μικρός*) and macro- (*μακρός*) and keeps the alliteration intact: micro-, macro-, and meta-heterogeneity. In the following section we will highlight several remarkable examples of observed meta-heterogeneity in protein glycosylation as reported in the literature, including several of our own very recent observations.

### Examples of reported meta-heterogeneity in protein glycosylation

There is likely no better example to start with than immunoglobulin G (IgG), perhaps the most-studied glycoprotein in terms of glycosylation to date. Not only is the protein an important adjustable player in the adaptive immune system and therefore the focus of a sizable multibillion-dollar pharmaceutical industry but also the *N*-glycosylation of the fragment-crystallizable (Fc)-region, Asn180 (IgG1 Uniprot nomenclature), is an important determinant of the protein's effector function (92). Overall, the typical glycan found on the site is a diantennary species with core fucose, 0 to 2 galactoses, trace levels of siaylation, and potential bisection (92). It is known that the absence of the fucose can increase binding to the FcγIII receptor and subsequent antibody-mediated cellular cytotoxicity by an order of magnitude (92), whereas the level of galactosylation appears to mediate complement-dependent cytotoxicity (93). In terms of meta-heterogeneity, however, it has to be noted that fully formed antibodies always have two disulfide-linked heavy chains and therefore two Asn180 *N*-glycosylation sites (93). It is currently not known whether the drastic increase in antibody-mediated cellular cytotoxicity requires the absence of both the two fucoses on each heavy chain, or whether one is enough. Similarly, through extensive glycoengineering of IgG1-producing B-cells near-full fucosylation, galactosylation, and sialylation could be achieved, but at the maximum just up to 50% bisection (93). This limit in bisection could have a technical reason or could be the enzymatic equilibrium, but it could also be that each IgG can only carry one bisection at most and 50% of the glycans represents 100% of the antibodies. This question can only be answered if the interaction between the two glycosylation sites on IgGs—the meta-heterogeneity of the glycosylation—is taken into consideration.

Next to Fc-glycosylation, it has been postulated that, in serum, up to 20% of circulatory IgG1s may carry an additional *N*-glycosylation site within their fragment antigen-binding (Fab) region (94, 95, 96). This additional *N*-glycosylation site may be generated by the recombination and hypermutation of the variable region, and the processing or selection of these antibodies may even be favored in certain disease phenotypes (96). One example of this is a group of autoantibodies commonly found in rheumatoid arthritis, the so-called anti-citrullinated protein antibodies. Not only were these, and their Fab-glycosylated variants, more often detected in patients with rheumatoid arthritis compared with controls (96), but also the increase in Fab-glycosylation precedes the development of clinical symptoms and might serve as an early-detection biomarker (97). In terms of meta-heterogeneity, Fab glycans differ notably from their Fc counterparts by exhibiting high levels of galactosylation, sialylation, and bisection (98). The exact reasons for this difference in glycan composition remain to be elucidated, but one can imagine that the unhindered Fab glycans represent the full glycan processing potential of the B cell, whereas Fc-glycans are sterically hindered within the limited space between the heavy chains and therefore only support limited and select modifications. Glycan meta-heterogeneity is large enough, however, that in mixtures of released glycans (in which knowledge about the glycosylation site is lost) the largest glycans can be presumed to originate from the Fab regions, facilitating rapid biomarker analysis by, *e.g.*, MALDI-TOF-MS (99, 100).

Another intriguing antibody in terms of meta-heterogeneity in protein glycosylation is immunoglobulin M (IgM), usually the first antibody to arrive (in penta- or hexameric form) at sites of infection and inflammation to activate the complement system (94, 101). Each heavy chain of IgM contains five *N*-glycosylation sites, namely, Asn46, Asn209, Asn272, Asn291, and Asn440. However, even though these sites are exposed to the same environment during glycan processing in the endoplasmic reticulum and Golgi apparatus, Asn46, Asn209, and Asn272 end up with complex *N*-glycans akin to those found on IgG1, while Asn291 and Asn440 carry high-mannose species instead (101, 102). These latter high-mannose glycans are localized at the penta-/hexameric center near the J-chain, whereas the complex glycans lie further to the outside in the direction of the Fab regions. In principle, high-mannose glycans require fewer enzymatic steps than complex glycans, and their presence may suggest hindrance or shielding at an early stage of processing (103). The net result is, however, that IgM can activate the complement system not only *via* the classical pathway (C1-complex), but owing to the high-mannose glycans at these specific sites also *via* the lectin pathway (mannose-binding lectin) (101, 104, 105), a clear example of meta-heterogeneity linked to specific function.

Immunoglobulin A1 (IgA1) is yet another antibody with interesting glycan meta-heterogeneity. This protein typically resides in the blood as a monomer or in human milk or mucosal tissue as a J-chain-linked dimer or even higher oligomers (106, 107). Next to abundant *O*-glycosylation at the hinge region, IgA1 is *N*-glycosylated at two positions on the heavy chain, namely, Asn144 and Asn340 (106, 107). Although both of these sites primarily carry complex diantennary glycans with high levels of galactosylation, sialylation, and partial bisection, it was also shown that the glycan at Asn340 is nearly always core-fucosylated, whereas in sharp contrast, Asn144 not at all (108, 109). The cause or functional consequences of this are not yet clear, but something must be preventing alpha-(1,6)-fucosyltransferase (FUT8) activity specifically on Asn144.

**Fig. 3:**
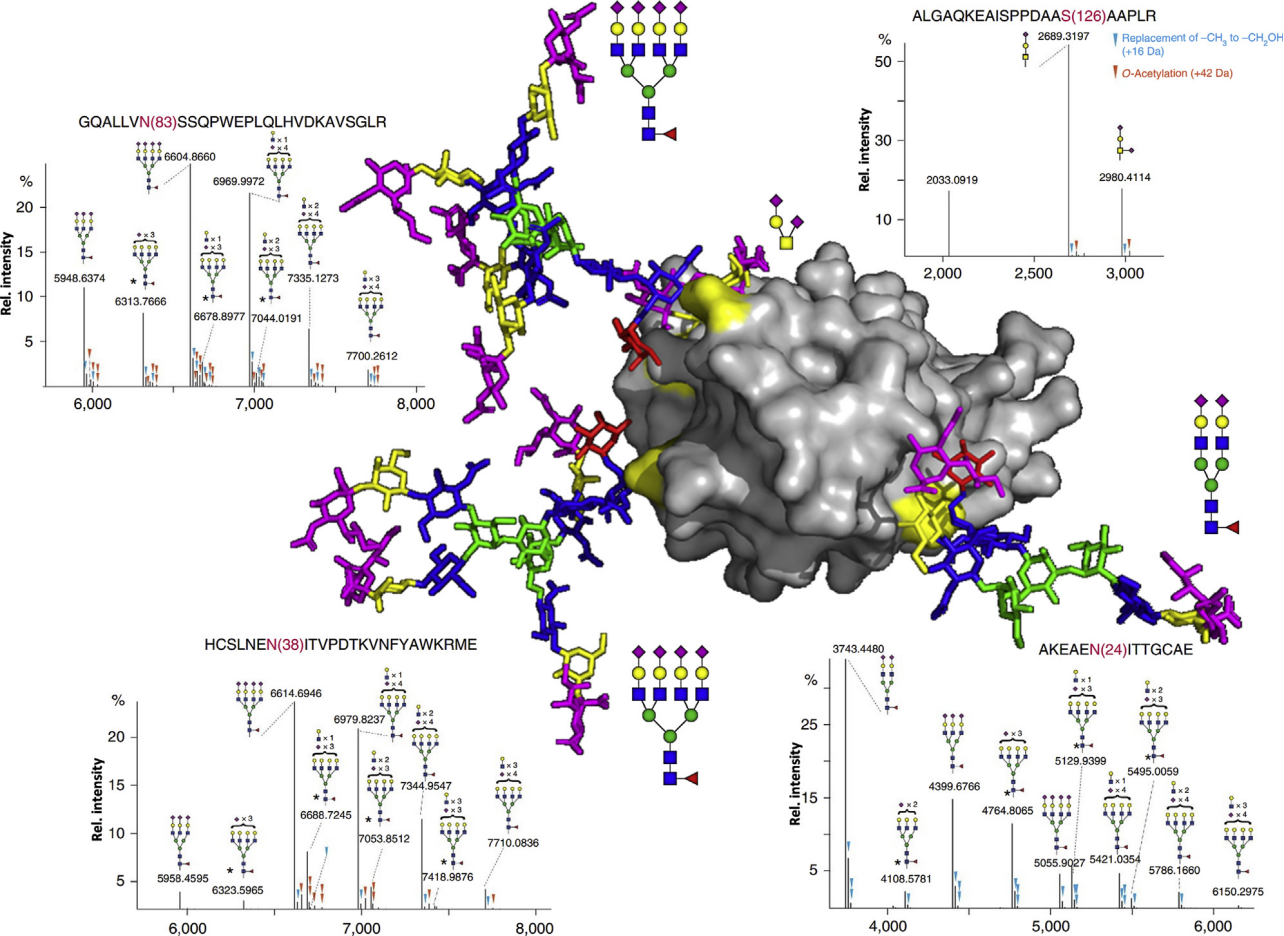
**Meta-heterogeneity in erythropoietin glycosylation**. Mass spectrometric analysis indicated that Asn83 (*top left*) and Asn38 (*bottom left*) predominantly carried tetra-antennary glycans and could contain *O*-acetylation, while the most abundant glycan at Asn24 (*bottom right*) was di-antennary for which no *O*-acetylation was found. Based on the structure there is no apparent steric hindrance that could explain the lower complexity of Asn24 glycosylation, suggesting a site-specific layer of regulation. The structure was modified from the PDB entry 1BUY (with Lys to Asn substitution), and all glycoprotein structures (throughout the review) were generated by means of GLYCAM (www.glycam.org) (112, 113). Note that the glycans are highly dynamic and will likely occupy a large region around the shown structure. The mass spectrometric data were reused with permission from Yang *et al.* (111).

Moving away from the immunoglobulins we next discuss erythropoietin (EPO), which is a glycoprotein cytokine secreted mainly by the kidney. EPO stimulates the production of red blood cells in the bone marrow, a feature that, next to incidental performance enhancement, can be medicative in cases of anemia (110). Because of this, the protein is produced as a therapeutic agent for which it is important to monitor batch-to-batch variation in both the protein backbone and the posttranslational modifications it harbors. EPO glycosylation comprises one *O*-glycosylation site and three *N*-glycosylation sites, the occupancy of which we recently investigated by a combination of bottom-up LC-MS/MS and native mass spectrometry (111). Although Asn24, Asn38, and As83 generally contained di-, tri-, tetra-, and even *N*-acetyllactosamine-extended glycosylation, we could also detect notable differences between recombinant EPO products rhEPO BRP, epoeitin beta, and epoetin zeta, mostly in terms of sialylation (111). One of the more surprising observations, however, was in the meta-heterogeneity of sialic acid acetylation (Fig. 3). *O*-Acetylation can occur at the hydroxyl groups of sialic acids (typically at the 4, 7, 8, or 9 positions) and is one of the characteristics that differentiates EPO between pharmaceutical suppliers (114). Interestingly, for the cases in which we detected *O*-acetylation, this only occurred at Asn38 and Asn83, and not at Asn24 (Fig. 3). Asn24 was also the site to show the largest portion of relatively small glycans, namely, diantennary, suggesting a limited accessibility to enzymes that may also encompass the sialyl *O*-acetyltransferase responsible for *O*-acetylation (111, 114). If so, this meta-heterogeneity needs to be kept in mind when engaging in recombinant EPO glycoengineering to optimize pharmaceutical parameters (115). Glycans on Asn38 and Asn83 of EPO require the presence of tetra-antennary *N*-glycans to cover the hydrophobic patch surrounding those sites, ensuring proper solubility in blood and preventing protein aggregation (116). This is also in agreement with our own observations wherein we expressed EPO in CHO cells glycoengineered to produce homogenous glycoforms (115). We noticed that, upon enzymatic deglycosylation (PNGase F) EPOs bearing tetraantennary *N*-glycans tended to aggregate and were challenging to measure, whereas the homogenous diantennary EPOs were amenable to deglycosylation and straightforward to analyze. This likely indicated that the structure of EPO modified with diantennary *N*-glycans undergoes slight changes in order to cover known hydrophobic patches (115).

Acute phase proteins generally express diantennary glycans with high levels of galactosylation and (α2,6-linked) sialylation, while increasing their antennarity, α2,3-linked sialylation, and antennary fucosylation upon acute inflammation (102, 117). The extent to which this occurs, however, depends highly on the precise nature of the glycosylation site. For alpha-1-antitrypsin, for example, it was reported that, among the three *N*-glycosylation sites, Asn107 may carry di-, tri-, and tetraantennary glycans, while Asn70 and Asn271 are primarily glycosylated with diantennary species (118). Similarly, the pentuply *N*-glycosylated alpha-1-acid glycoprotein displays its largest tetraantennary glycans at sites Asn72, Asn93, and Asn103, while the glycans at Asn33 and Asn56 do not exceed three antennae (119, 120).

**Fig. 4:**
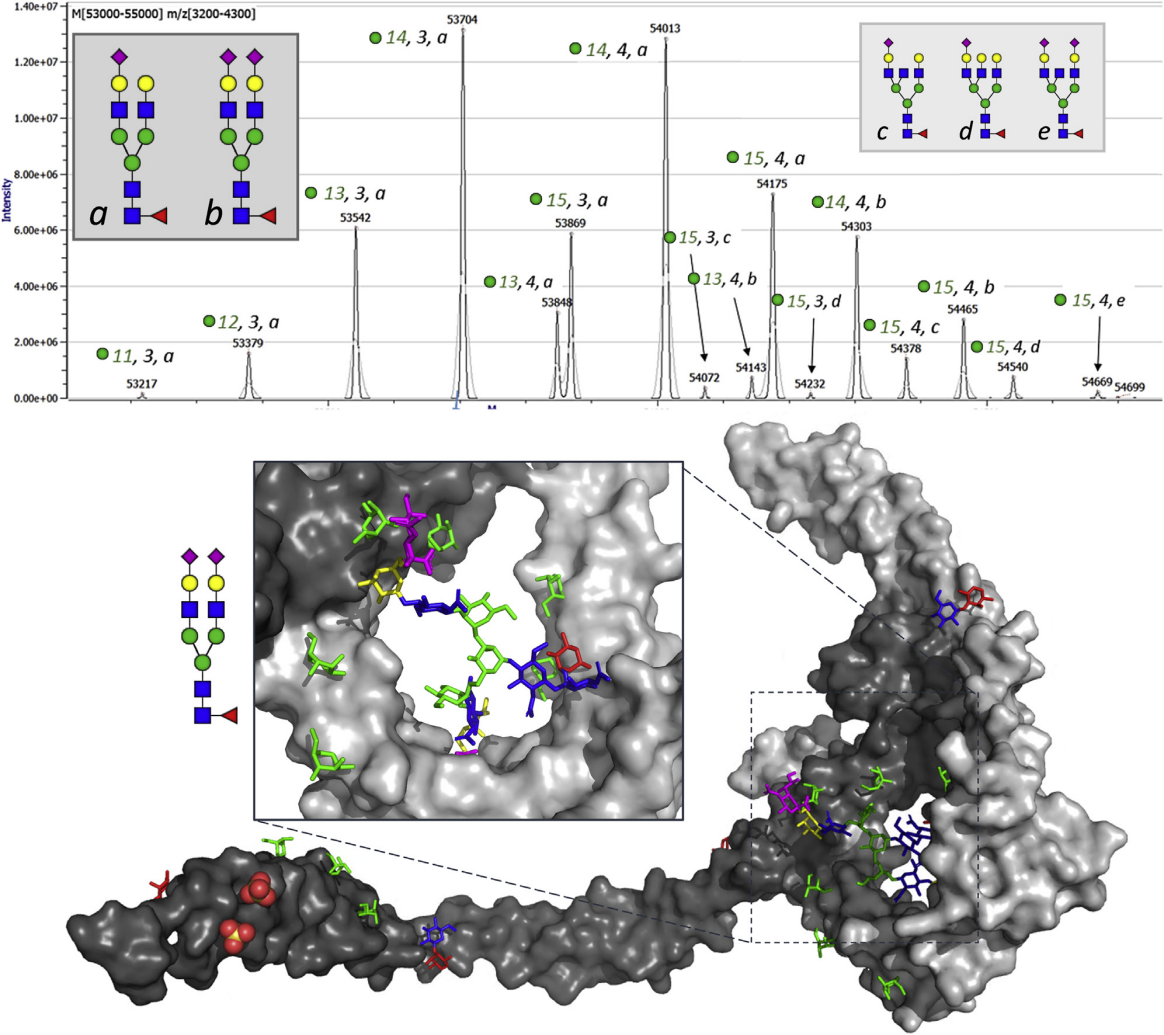
**Meta-heterogeneity cross talk found between properdin (complement factor P) *N*-glycosylation and *C*-mannosylation**. As seen from the deconvoluted native mass spectra of properdin (*top*), diantennary *N*-glycoforms (*A* and *B*) co-occur with 11 to 15 *C*-mannosylations, while triantennary N-glycoforms (*C–E*) only co-occur with 15 *C*-mannosylations. In the properdin structure (*bottom*) (6RUS) (123) it can be seen that part of the *C*-mannosylation occurs in the same region as the *N*-glycan, making cross talk conceivable. One explanation may be that full *C*-mannose occupancy hinders the *N*-glycan from entering the protective pocket, thereby exposing the sugar for more extended modification by glycosyltransferases. Th mass spectrometric data were reused with permission from Bern *et al.* (122).

Properdin, a plasma protein also known as complement factor P, has a critical role in complement activation *via* the alternative pathway (121). We profiled the proteoform profile of properdin in order to gain insight into the possible role of post-translational modifications into the protein's biological functions (111, 122). By combining data from native mass spectrometry and intact glycopeptide analysis, we were able to fully characterize the protein's PTM profile (111, 122). Our analysis revealed a single *N*-glycosylation site, 4 *O*-glycosylation sites predominantly carrying a GlcFuc disaccharide, and 17 Trp residues that could be modified with *C*-linked mannosylation. However, despite the myriad of potential modifications, the protein actually exhibited small heterogeneity in its glycosylation. As seen from the native spectrum (Fig. 4), the majority of properdin molecules was modified with 13 to 15 *C*-mannosylations, 3 to 4 *O*-glycosylations, and a di- or triantennary *N*-glycan. However, we also stumbled upon a surprising discovery in terms of meta-heterogeneity: triantennary *N*-glycans were found concurrently with 15 *C*-mannosylations, whereas only diantennary *N*-glycans could be found on molecules with 13 or 14 *C*-mannosylations (Fig. 4). Explanations for this co-occurrence of posttranslational modifications are speculative, but one option may be that the accessibility or flexibility of the protein is adjusted by the *N*-glycan size, while it may also be that the distinct glycoforms originate from different cell types.

With a molecular weight of approximately 550 kDa, apolipoprotein B-100 (ApoB-100) is probably the largest protein involved in lipoprotein assembly (*i.e.*, into low-density lipoprotein and very low-density lipoprotein particles), and consequentially an important player in the plasma transport of phospholipids, triacylglycerol, and cholesterol (124). The amino acid sequence of ApoB-100 contains 19 *N*-glycosylation sites, and remarkable meta-heterogeneity is observed in the occupying glycan species. Complex (mostly sialylated diantennary) glycans were found at Asn983, Asn2239, Asn2779, Asn2982, Asn3101, Asn3224, Asn3465, Asn3895, Asn4237, and Asn4431, while high-mannose glycans occupied Asn185, Asn1368, Asn1377, Asn3336, and As3358 (125). Furthermore, both high-mannose and complex glycans were shown to be possible at Asn1523 and Asn3411, and no glycosylation has yet been found at Asn34 and Asn2560 (125). As a structure, the long ApoB-100 sequence winds through the lipoprotein's membrane phospholipids, allowing for glycosylation sites that face the extracellular side, those that are close to the membrane, and those that face the lipophilic lumen. It is conceivable that the meta-heterogenous glycosylation is a consequence or even the cause of this particular organization, but this question is still open for debate.

**Fig. 5:**
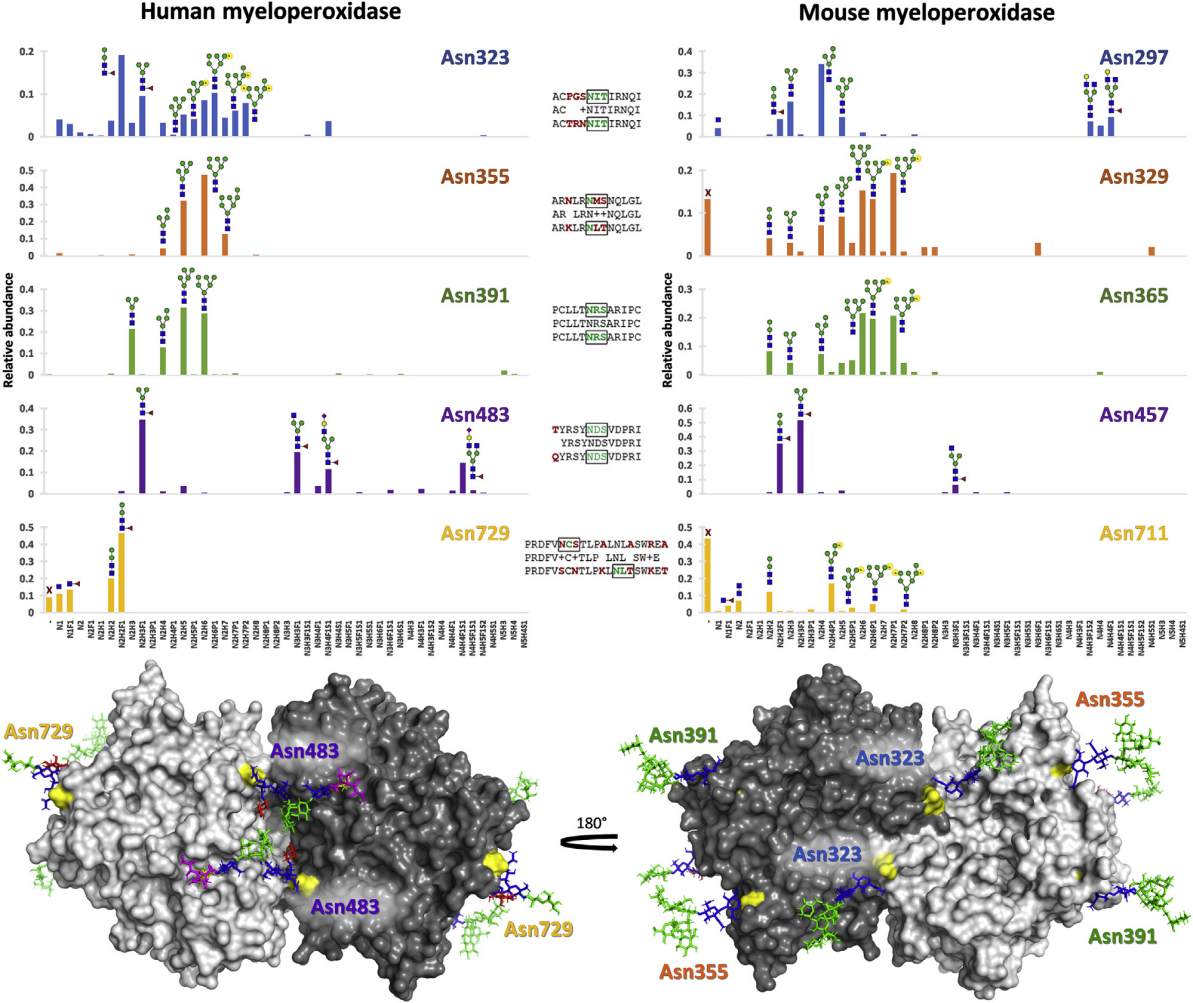
**Similarities and differences in glycan meta-heterogeneity between human and mouse myeloperoxidase (MPO).** Human MPO (*top left*) is a highly meta-heterogenous glycoprotein with phosphomannosylation at Asn323, high-mannose species at Asn355 and Asn391, complex species at Asn483, and paucimannosylation/macro-heterogeneity at Asn729. Mouse MPO (*top right*) shows distinct differences in this meta-heterogeneity, for example, by carrying phosphomannose species at Asn329, Asn365, as well as Asn711. The structure of dimeric MPO (*bottom*) was generated from the human database (1CXP) (91) and shows representative glycosylation. The mass spectrometric data were reused with permission from Reiding *et al.* (90).

Recently, we reported the in-depth characterization of the human neutrophil protein myeloperoxidase (MPO), a potent antimicrobial agent, in which we applied a combination of bottom-up LC-MS/MS and native MS to fully capture the protein's glycan heterogeneity in a site-specific manner (90). Next to exhibiting uncommon glycosylation characteristics such as pauci- and phosphomannosylation, the protein turned out to be a particularly striking example of meta-heterogeneity; of the five glycosylation sites, Asn391 showed high-mannose species with low variation between donors, Asn355 showed either high-mannose or paucimannose species depending on the donor, Asn483 showed complex glycosylation, Asn323 showed large amounts of phosphomannosylated glycans, and Asn729 either showed truncated glycans down to a single *N*-acetylglucosamine or was unoccupied altogether (90). We performed the same analysis on murine MPO from WEHI 3BD cells (granulocytic leukemia from the mouse strain BALB/c) and could replicate the large fractions of pauci- and phosphomannose glycosylation (Fig. 5), meaning that these atypical phenotypes are not exclusive to human proteins but rather occur across species. The observed meta-heterogeneity, however, differed considerably between human and mouse MPO. Although human MPO only displayed one site with phosphomannosylation (Asn323), mouse MPO abundantly showed the trait on Asn329 (equivalent to human Asn355), Asn365 (Asn391), and Asn711 (Asn729). Next to this, mouse MPO showed smaller glycans in general for each of the sites, no sialylation (either *N*-acetylneuraminic acid or *N*-glycolylneuraminic acid), and abundant macro-heterogeneity not only on Asn711 (Asn729) but also on Asn329 (Asn355) (Fig. 5). We presume the pauci- and phosphomannosylation of MPO to occur because the protein is generally stored in neutrophil azurophilic granules, which share many characteristics with the lysosomes that can be found in other cell types (126). Phosphomannosylation may then serve a function akin to lysosomal trafficking, meaning that phosphomannosylated proteins could preferentially end up in the azurophilic granules (127). Paucimannose species may be the result of the many glycosidases present in the granule, including alpha-mannosidases and beta-hexosaminidases (128). If so, given that both human and mouse MPO still display large glycans at several of their glycosylation sites must mean these glycans were protected against degradation, possibly by interactions with the peptide backbone. Crucially, the difference in meta-heterogeneity between human and mouse MPO suggests that these glycan-protein interactions are not the same between species, which should be kept in mind in future studies.

We conclude with the envelope protein (Env) of human immunodeficiency virus 1 (HIV-1), the target of broadly neutralizing antibodies that were shown to protect against virus challenges in animal models (129, 130). Env is extensively glycosylated with 26 to 30 *N*-glycosylation sites and is naturally present as a trimer comprising up to 90 *N*-glycans. Together, the *N*-glycans form a glycan shield that is recognized by the broadly neutralizing antibodies, meaning that knowledge of the site-specific glycosylation is of the utmost importance for vaccine development (131, 132, 133). A recent detailed analysis of 11 HIV-1 Env proteins (including both bound and soluble forms of the trimer) differing in the construct design, purification methods, and cell line of origin (134) uncovered an incredible meta-heterogeneity of glycosylation on the protein: Asn156, Asn262, Asn344, Asn389 to Asn488, and Asn465 were occupied exclusively with high-mannose *N*-glycans in all trimers, while sites Asn187, Asn197, Asn230, Asn234, Asn356, and Asn386 presented more processed complex glycoforms. Other sites instead showed differences in glycosylation between the 11 HIV-1 Env trimers and generally proved more variable. Another recent study uncovered the differences in meta-heterogeneity between HIV envelope glycans and a recombinant trimer that was soluble (135). In this work, most cases of membrane-bound and virus-bound Env displayed a higher level of complex-type *N*-glycans when compared with recombinant soluble Env trimer, which comparatively carried more high-mannose *N*-glycosylation. One striking glycosylation site (Asn301) differed entirely between the soluble and membrane-bound versions, respectively, carrying a high-mannose or complex *N*-glycan. Altogether, with these highly glycosylated virus proteins it will be the dense combination of glycans that is presented to the immune system rather than single glycans from single sites, meaning that the glycan meta-heterogeneity will be an important factor to consider to tailor the immune system against complex viruses like HIV-1.

## Conclusion and future perspectives

In this review we introduced the term meta-heterogeneity to complement the already existing terms used to describe variation in protein glycosylation, *i.e.*, micro-heterogeneity (variation in glycosylation) and macro-heterogeneity (variation in glycan occupancy). With the steady maturation of mass spectrometric methodologies, including improvements in fragmentation methods, acquisition speed, and the availability of data analysis tools, large-scale glycoproteomics has become feasible. This has enabled not only insights into biological roles of glycosylation but also the observation of differences and interactions between glycans on proteins with multiple glycosites. For this reason, we deemed it useful to introduce the aforementioned new term. In this review, we aimed to cover distinct literature examples of meta-heterogeneity, thereby discussing various immunoglobulins, erythropoietin, and various serum and plasma proteins, as well as the neutrophil-specific protein myeloperoxidase. It has to be noted, however, that this represents only a fraction of the available information and that in our observation most glycoproteins exhibit one form of meta-heterogeneity or another.

When interpreting the reasons for meta-heterogeneity we have to consider that the overall glycosylation of a protein is dependent on a number of factors. These include (1) the production capabilities of a parent cell, as glycosidases, glycosyltransferases, and nucleotide sugars need to be expressed and present in the endoplasmic reticulum/Golgi apparatus to build glycosylation characteristics (89, 103); (2) the directed extracellular modification of protein glycosylation, *e.g.*, by platelet galactosyl- and sialyltransferases (136), or detrimentally by pathogens such as by influenza neuraminidase action (137), and (3) the glycan-dependent clearance mechanisms, *e.g.*, hepatocyte asialoglyoprotein receptors (138), Golgi apparatus or cell surface mannose-6-phosphate receptors (127), or dendritic cell/macrophage mannose receptors (139, 140, 141). However, the presence of high glycan meta-heterogeneity—having differing glycan species at different glycosylation sites of the same protein—necessitates site-specific mechanisms as well. Investigations into site-specific glycan processing have been sparse, but one can, for example, think of steric hindrance, chaperoning, compartmental partitioning, or extended glycosylation sequons. One could also imagine that meta-heterogeneity enables the encoding of multiple biological functionalities within the same protein, based on the differing glycan epitopes found on different glycosylation sites.

Looking into the future, it has already become apparent that to fully map protein meta-heterogeneity glycopeptide analysis is not sufficient, but it is also necessary to obtain a proteoform profile of the intact protein with its glycosylation. A method with most promise in this area is high-resolution native mass spectrometry. As demonstrated here on the properdin case, only with native MS could we observe the presence of triantennary glycans exclusively on properdin carrying 15 *C*-mannosylations, something that could not be observed at the glycopeptide level. Similarly, in order to elucidate the biological functions of meta-heterogeneity, such as influencing drug binding or protein interactions, the combination of glycoproteomics and native MS is crucial (142, 143).

In all, we believe it will be increasingly important to not just regard glycosylation on a site-to-site basis but rather as a whole across glycoproteins and glycoprotein complexes. We hope that the term meta-heterogeneity will help us to efficiently conceptualize this.

## Conflict of interest

Authors declare no competing interests.
